# Resilient virtual inertia strategy for frequency support of renewable-based microgrids using a variable structure fuzzy PID controller

**DOI:** 10.1038/s41598-026-43661-y

**Published:** 2026-03-31

**Authors:** M. A. Abdelghany, Gaber Magdy, A. M. Abdel Ghany, Fatma A. Mohammed

**Affiliations:** 1 Electrical Engineering Department, Faculty of Engineering, October 6 University, Giza, Egypt; 2 Department of Electrical Engineering, Faculty of Energy Engineering, Aswan University, Aswan, 81528 Egypt; 3 Faculty of Engineering, King Salman International University, El-Tor, 46511 Egypt; 4 Electrical Power and Machines Department, Faculty of Engineering, Helwan University, Cairo, Egypt; 5 El Safwa Higher Institute of Engineering and Technology, El Shorouk, Egypt

**Keywords:** Variable structure control, Fuzzy logic control, Frequency stability, Virtual inertia control, Renewable energy, Microgrid, Energy science and technology, Engineering, Mathematics and computing

## Abstract

Ensuring frequency stability in low-inertia microgrids with high penetration of renewable energy sources (RESs) is a critical challenge, as these sources lack physical inertia and generate variable, unpredictable power. This leads to amplified frequency deviations, further aggravated by nonlinearities and stochastic behavior. Virtual inertia control, which emulates the inertial response of synchronous generators using energy storage, has emerged as a promising solution. However, conventional approaches often lack the responsiveness and robustness required to manage RES uncertainties and system nonlinearities, thereby limiting their effectiveness in dynamic environments. Moreover, the application of variable structure fuzzy logic controllers for load frequency control in renewable-dominated, low-inertia microgrids remains largely underexplored. To address these limitations, this paper proposes a variable structure fuzzy proportional–integral–derivative (VSC-FPID) controller designed for virtual inertia support in low-inertia microgrids. The proposed controller is tested under diverse loading scenarios and nonlinear conditions to ensure comprehensive evaluation. Its parameters are optimally tuned using the particle swarm optimization algorithm to guarantee efficient and robust dynamic performance. Comparative analysis with conventional PID and fuzzy-PID controllers demonstrates the superior capability of the VSC-FPID controller. Simulation results conducted in MATLAB confirm its robustness in regulating frequency under load fluctuations, RES uncertainties, and nonlinear system dynamics, including severe low-inertia and worst-case operating conditions involving simultaneous load and renewable disturbances. The proposed controller consistently outperforms benchmark methods, significantly improving transient and steady-state performance. Specifically, it achieves notable reductions in overshoot, undershoot, and settling time, with up to 60% improvement compared to the nearest competing technique. These results highlight the potential of the VSC-FPID-based virtual inertia control strategy as an effective solution for enhancing frequency stability in renewable-rich, low-inertia microgrids.

## Introduction

 Renewable energy sources (RESs), including solar photovoltaic (PV) and wind power, have increasingly become fundamental to the structure and operation of modern power systems. This shift toward renewables offers notable economic advantages and environmental benefits. However, the fluctuating and weather-dependent nature of RESs present significant challenges to system stability, as these sources are non-dispatchable and dependent on weather conditions^1^. A direct consequence of increased RESs integration is significant reduction in system inertia, which traditionally comes from conventional synchronous generators. This decline in inertia, combined with fluctuating renewable generation output, results in persistent imbalances between generation and demand. Such imbalances lead to continuous variations in system frequency and voltage. In low-inertia systems, the reduced stored kinetic energy limits the system’s ability to naturally counteract power mismatches, causing frequency deviations to persist for longer durations rather than being quickly damped. Moreover, the inverter-based nature of RESs decouples generation dynamics from grid frequency, further weakening the inherent self-regulating capability of the system. As a result, frequency stability becomes highly sensitive to load variations and renewable intermittency, necessitating advanced control strategies capable of actively compensating for inertia deficiency. Moreover, with reduced inertia, these frequency deviations tend to be more pronounced and occur more rapidly, increasing the risk of severe frequency instability. These frequency fluctuations have detrimental effects on both generation units and electrical loads, potentially exposing equipment to damaging conditions such as deep frequency nadirs and excessive rates of change of frequency (RoCoF). If not properly managed, these disturbances can interfere with system protection schemes, leading to undesirable outcomes like load shedding, generator tripping, or even cascading outages^2^. On the other hand, some existing power systems have a multi-source generating system including reheat and non-reheat thermal units, hydroelectric stations, and RESs^3^. Each of these generation sources introduce distinct nonlinear dynamic behaviors, including governor deadband (GDB) and generation rate constraints (GRC), which complicate system modeling and control. When combined with the stochastic nature of RESs and fluctuating load demand, these factors highlight the critical need for an advanced and highly effective load frequency control (LFC) strategy capable of maintaining frequency stability under diverse operating conditions.

The large-scale integration of inverter-based RESs, such as wind and solar PV systems, fundamentally alters power system dynamics by displacing conventional synchronous generators that inherently provide rotational inertia. As a result, modern microgrids experience a substantial reduction in effective system inertia, which weakens their natural ability to counteract power imbalances between generation and demand^2^. In low-inertia environments, even small disturbances can lead to pronounced frequency deviations, elevated rates of change of frequency (RoCoF), and prolonged transient responses^3^. These effects are further exacerbated by the stochastic nature of RES generation and nonlinear system characteristics, leading to persistent frequency imbalances that cannot be effectively mitigated using conventional fixed-parameter control strategies. Consequently, advanced control approaches capable of actively compensating for inertia deficiency and dynamically adapting to operating conditions are essential for ensuring frequency stability in renewable-dominated microgrids.

LFC’s goals are to reduce transient variances in frequency while ensuring zero steady-state error. According to recent research, a wide range of techniques have been applied within LFC loops across different power systems to meet these goals. The frequency support loops are classified into primary frequency control which acts within 30 s after disturbance and mainly is performed by governor action in the conventional stations, the secondary controller, which restores frequency to its nominal value by adjusting the governor setpoint, and supplementary control, which employs external energy storage systems (ESSs) such as batteries, flywheels, or superconducting magnetic energy storage (SMES) units to enhance system stability^4’5^. The use of supplementary frequency support significantly enhances system response, with further improvements achievable by increasing the capacity of the ESS. However, this enhancement entails additional investment costs associated with the deployment of larger energy storage devices. In^6^, the authors proposed a robust LFC strategy that leverages electric vehicles as distributed energy storage units. Meanwhile, fractional-order (FO) proportional-integral (FOPI) and FO proportional-derivative (FOPD) controllers were introduced in^7^ to enhance LFC performance in multi-source power systems. Additionally, a two-degree-of-freedom proportional–integral–derivative (2-DOF PID) controller has been implemented for frequency regulation in a multi-area interconnected power system, demonstrating enhanced performance in handling dynamic disturbances^8^. While conventional linear controllers can perform adequately under nominal conditions, their effectiveness diminishes notably when system nonlinearities and shifting operating points come into play. To address these nonlinear dynamics and maintain reliable performance across varying conditions, the adoption of nonlinear or robust control strategies becomes essential. Fuzzy logic controllers represent a class of nonlinear controllers known for their simple design, ease of implementation, and ability to handle system nonlinearities effectively. Despite these advancements, most existing LFC strategies are designed with fixed controller structures that are unable to adapt their control behavior in response to rapid operating condition changes. This limitation becomes particularly critical in low inertia microgrids, where system dynamics vary significantly due to renewable intermittency and nonlinear effects.

Various types of fuzzy logic algorithms have been developed for control applications. The conventional Mamdani fuzzy controller commonly operates as a fuzzy-PD controller^9^. However, to address steady-state errors more effectively, fuzzy PI or fuzzy PID controllers are often preferred. In^10,11^, researchers proposed fuzzy PID controllers specifically tailored for the LFC. In^12^, a Type-II fuzzy PID controller was developed to enhance the frequency regulation in a complex diverse-source power system, incorporating both a unified power flow controller and SMES. Additionally, a self-tuned fuzzy PI controller tailored for LFC applications was presented in^13^. Fuzzy logic has also been employed for the real-time adjusting of conventional PID controller parameters, as demonstrated in^14^. In^15^, a FO fuzzy-PID controller was implemented for frequency regulation of an interconnected power system. Despite the extensive use of fuzzy logic–based controllers in LFC applications, the majority of existing studies rely on fixed-structure fuzzy PI or fuzzy PID controllers with static rule bases and invariant membership functions. Such fixed-structure designs inherently limit adaptability under rapidly changing operating conditions, particularly in low-inertia microgrids characterized by high renewable energy penetration and strong nonlinearities. Although variable structure fuzzy logic controllers have been investigated in other power system applications, such as power system stabilizers and wind turbine yaw control, their systematic application to LFC, especially within a virtual inertia–based framework, remains largely unexplored. This research gap motivates the development of the proposed variable structure fuzzy PID (VSC-FPID) controller, which dynamically adapts its control behavior in real time to enhance frequency regulation performance under renewable intermittency, load disturbances, and inertia uncertainty.

Motivated by the aforementioned observations, this paper proposes a variable structure control for a fuzzy PID controller (VSC-FPID) to achieve robust secondary frequency regulation within the LFC framework. As highlighted in^4^, current variable structure controllers applied to LFC continue to encounter challenges in tuning and optimization—challenges that are exacerbated in power systems incorporating variable RESs such as wind and solar PV. Although variable structure fuzzy logic controllers have been investigated and deployed in single-area and multi-machine systems primarily as power system stabilizers^16^, and in yaw control mechanisms for wind turbines^17^, their specific application to LFC remains underexplored. This gap in the literature underscores the novelty and significance of the proposed VSC-FPID controller in addressing modern grid control requirements. To the best of the authors’ knowledge, this work represents one of the first systematic investigations of a VSC-FPID controller integrated within a virtual inertia-based LFC framework for low-inertia microgrids.

To fully exploit the capabilities of the suggested VSC-FPID controller, its adjusting parameters must be meticulously chosen, with particular attention given to the nonlinear nature of the power system. In recent years, meta-heuristic optimization algorithms have become widely adopted for tuning controller parameters due to their effectiveness in handling complex and nonlinear optimization problems. For instance, in^18^, a nonlinear PI controller-based dandelion optimizer has been proposed for frequency stability improvement of hybrid power grid. In^19^, a PID-based LFC scheme was optimized using the Runge-Kutta algorithm for a real interconnected power system. Meanwhile, Harris Hawks Optimization was utilized in^20^ to fine-tune a PI controller for effective frequency regulation in a two-area interconnected power system. Similarly, in^7^, the dragonfly search algorithm was employed to optimize the parameters of a cascaded FOPI–FOPD controller for LFC in a multi-source power system. In^9^, the artificial bee colony algorithm was utilized to design and tune various fuzzy controllers, including fuzzy PID, PI, PD, and conventional fuzzy logic controllers. Additionally, in^12^, the water cycle algorithm was applied to optimize a fuzzy PID controller for enhancing the LFC performance of an interconnected power system. Among the various optimization techniques, particle swarm optimization (PSO) has gained significant attention due to its fast convergence, ease of implementation, and robustness. In this study, PSO is adopted to optimally tune the parameters of the proposed controller. The objective function for the optimization process incorporates key dynamic performance criteria, such as minimizing the frequency nadir, reducing oscillations, and enhancing system recovery speed following load disturbances.

To evaluate the performance of the proposed VSC-FPID controller, it is applied to an islanded microgrid, serving as a representative case study. The proposed controller’s performance is assessed through a series of comprehensive simulations under diverse operating conditions, including step load power (SLP) changes, stochastic load variations, and scenarios with both industrial and residential load profiles. Furthermore, the microgrid incorporates solar and wind generation units, with the analysis considering the unplanned disconnection and reconnection of these renewable sources. The proposed controller’s robustness is further examined under conditions of reduced system inertia. The performance outcomes are benchmarked against those obtained from an optimally tuned PID controller and an optimally tuned fixed-structure fuzzy PID controller. The key contributions of this study can be summarized as follows:

i. Propose a resilient virtual inertia control strategy based on a VSC-FPID to support the frequency stability of an islanded microgrid with high RESs penetration.
ii. A well-established optimization technique, The PSO algorithm, is employed to fine-tune the parameters of the proposed controller within the virtual inertia control loop, thereby improving frequency regulation in the studied microgrid.
iii. The design process of the proposed controller explicitly accounts for system nonlinearities, such as GRC and GDB, as well as the uncertainties associated with RESs and load variations.
iv. A comprehensive performance evaluation is conducted by comparing the proposed controller with optimally tuned PID and fuzzy PID (FPID) controllers under diverse disturbances, including random load variations, unpredictable fluctuations in RESs, and system parameter uncertainties.

Therefore, this work provides one of the comprehensive investigations of variable structure fuzzy PID controllers for virtual inertia–based load frequency control in renewable-dominated, low-inertia microgrids.

The paper is organized as follows: Sect.  2 outlines a comprehensive description of the studied microgrid model. Section  3 elaborates on the proposed variable structure fuzzy controller and the adopted tuning methodology. Section  4 presents a thorough analysis of the controller’s performance under various operating disturbances, including load and renewable generation fluctuations. Section  5 summarizes the key conclusions and contributions of this study.

## Microgrid configuration and dynamic modeling

**Fig. 1:**
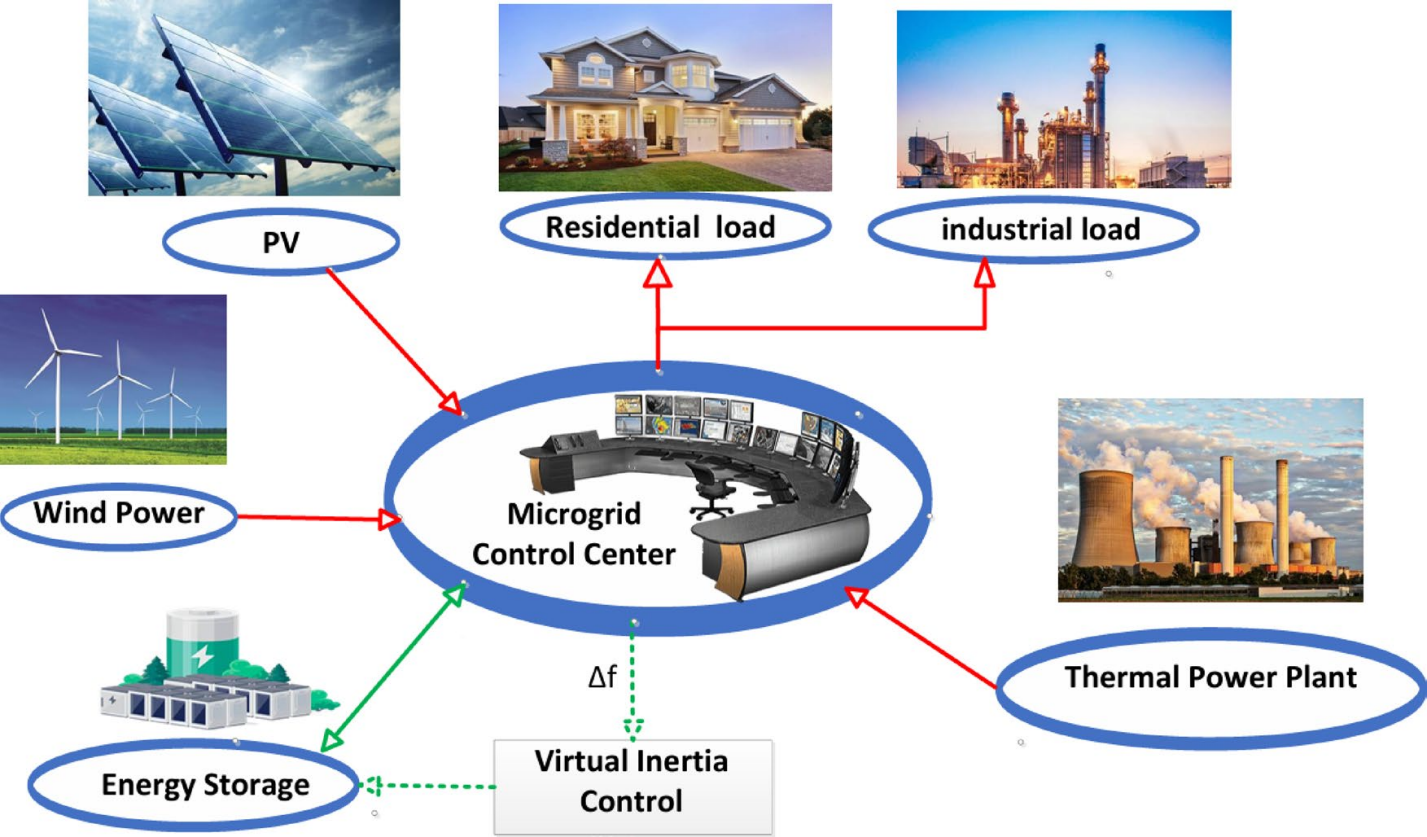
A simplified representation of the microgrid being studied.

The microgrid considered in this study, which incorporates RESs, is illustrated in Fig. 1. The system includes a diverse mix of generation and consumption resources, featuring 12 MW from a non-reheat thermal unit, 6 MW of solar PV output, 7 MW of wind energy, and 4 MW of energy storage, supplying power to 10 MW of industrial and 5 MW of residential demand. The system’s base capacity is 15 MW^21^. In this configuration, the thermal power unit provides both the required active power and primary frequency control. Secondary frequency control is handled through minimization of the Area Control Error (ACE), ensuring that steady-state frequency deviations are reduced. The ESS not only supports the thermal unit in supplying active power but also emulates virtual inertia and damping characteristics to enhance the microgrid’s frequency stability. Although solar and wind generation contribute significantly to the total power supply, they do not actively participate in frequency regulation. Consequently, the microgrid is subjected to disturbances caused by fluctuating wind and solar generation, along with varying residential and industrial loads. This modeling approach aligns with the methodologies presented in^22–26^.

The frequency behavior of the microgrid is described by the swing equation, as shown in Eq. (1), where the frequency deviation is represented by *∆f*. In this context, *∆P*_*m*_ denotes the total mechanical power supplied by conventional generation units, such as thermal power plants. Equation (2) similarly defines *∆P*_*RE*_ as the aggregate power contribution from RESs, specifically wind and solar PV systems.

1 $$\Delta {\mathrm{f}} = \frac{1}{{2Hs + D}}\left( {\Delta P_{m} + \Delta P_{{RE}} - \Delta _{L} } \right)$$

2 $$\varDelta{P}_{RE}=\varDelta{P}_{W}+\varDelta{P}_{PV}$$

Here, the parameters *H* and *D* represent the system’s inertia constant and damping coefficient, respectively. The subsequent subsections provide comprehensive descriptions of each microgrid component considered in this study.

A. **Non-reheat power plant**

The governor’s droop response of the thermal power plant is defined by Eq. (3), where $\varDelta{P}_{c}$ denotes the reference power setpoint, and *R* is the governor’s regulation factor. The governor’s dynamic behavior of the governor, including the effects of signal amplification and conversion, is represented by a first-order dynamic model characterized by a time constant ${T}_{g}$​, as expressed in Eq. (4).

3 $$\Delta P_{g} = - \frac{1}{{T_{g} s + 1}}(\Delta P_{{ACE}} - \frac{1}{R}\Delta f)$$

4 $$\varDelta{P}_{m}=\frac{1}{{T}_{t}s+1}\varDelta{P}_{g}$$

5 $$\Delta P_{{ACE}} = \frac{{K_{i} }}{S}(\beta .\Delta f)$$

B. **Wind generation system model**

As part of the wind power system, the turbine captures wind flow and converts it into mechanical torque. Although wind turbines generally operate at their maximum power point (MPP), the turbine speed must be continuously adjusted to ensure MPP operation under varying wind speeds. To simplify the complex dynamic response of transitioning between operating points, a first-order transfer function with a time constant ${T}_{WT}$​ is applied, as expressed in Eq. (6). In this equation, $\varDelta{P}_{wT}$ denotes the turbine’s output power. As demonstrated in the case studies, the turbine’s available power $(\varDelta{P}_{T})$ is calculated based on real-time wind speed and the specific characteristics of the turbine.

6 $$\varDelta{P}_{wT}=\frac{{K}_{WT}}{{T}_{WT}s+1}\varDelta{P}_{T}$$

C. **PV generation system model**

The solar PV system utilizes a PV array to convert solar energy into electrical power. To ensure optimal performance, the PV control system periodically scans the PV array’s power curve to maintain operation at the MPP. The dynamic behavior of the PV system can be approximated using a first-order transfer function characterized by a time constant ${T}_{pv}$​, as given in Eq. (7). In this equation, *∆Ps* denotes the solar input power, while $\varDelta{P}_{PV}$ corresponds to the output power produced by the PV array.

7 $$\Delta P_{{pv}} = \frac{{K_{s} }}{{T_{{pv}} s + 1}}\Delta P_{s}$$

**Fig. 2:**
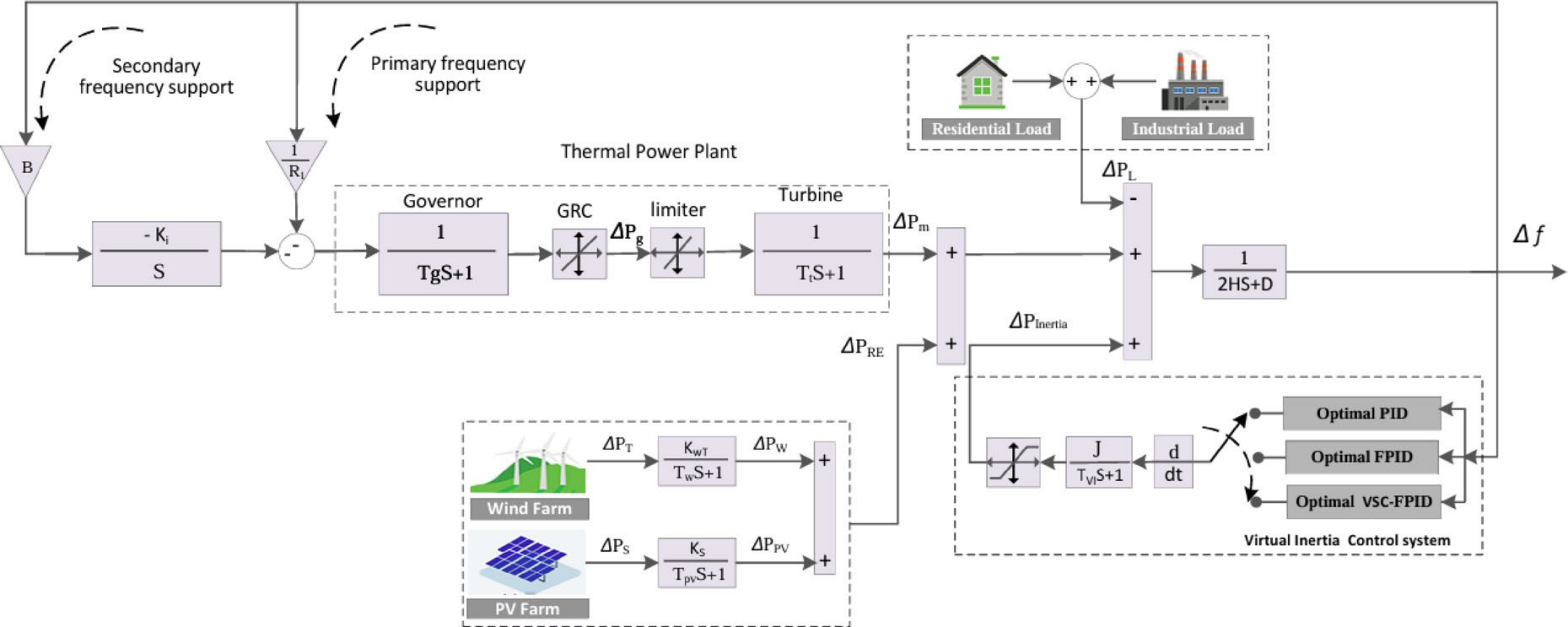
A dynamic model of the studied microgrid with the proposed virtual inertia control based on VSC-FPID.

Figure 2 depicts the dynamic representation of the examined microgrid. The non-reheat thermal generator operates under a generation rate constraint (GRC) of 12% per unit MW/minute. All associated system parameters are comprehensively listed in Table 1.

**Table 1:** Microgrid system parameters and their assigned Values^26^.

Parameter	Value
System inertia (*H*)	0.083 s
Droop characteristic, ( *R* )	2.4 Hz/pu
System damping coefficient, (*D*)	0.015 p.u MW/Hz
Time constant of the governor, (*T*_***g***_ )	0.1 s
Time constant of a turbine, (*T*_***t***_)	0.4 s
Time constant of ESS-based virtual inertia, (T_**VI**_)	10 s
Virtual inertia gain (*J*)	0.5
P_inertia Max_	0.25 pu
P_inertia Min_	-0.25 pu

## Proposed virtual inertia control technique

The proposed virtual inertia control strategy directly addresses inertia deficiency by emulating synchronous generator dynamics through ESS-based power electronic interfaces. Virtual inertia control is pivotal for strengthening the stability of modern power systems, particularly in microgrids. Its primary objective is to replicate the inertial response of conventional synchronous generators (SGs), thereby improving frequency regulation in systems with high penetration of RESs. Numerous control strategies have been proposed to improve the effectiveness of virtual inertia systems. SGs possess quite a lot of key characteristics that have warranted the reliable operation of electric power systems for years. One of the most vital features is the inertia energy kept in the rotating mass, which plays a crucial role in dropping the rate of change of frequency (RoCoF) afterward disturbances and pretty frequency stability. Nevertheless, RESs integrated to the grid concluded power electronic converters lack rotating mass, constructing them inherently low-inertia systems. By way of future power systems integrating an advanced portion of RESs, they are expected to exhibit lesser inertia constants, leading to significant frequency deviations and advanced RoCoF, which could result in frequency unsteadiness and potential blackouts.

To address this challenge, the concept of the virtual synchronous generator (VSG) has been introduced to enhance the apparent inertia of low-inertia power systems. This is achieved by embedding a dedicated control loop within the power electronic converters of ESSs, enabling them to mimic the dynamic behavior of conventional synchronous generators. However, due to the high cost associated with ESS deployment, large-scale implementation could result in substantial financial burdens. Alternatively, the rapid proliferation of electric vehicles (EVs), with a significant number expected to be deployed soon, presents a promising opportunity. While unmanaged EV charging can introduce operational challenges for the power grid, smart charging strategies can enable EVs to actively contribute to ancillary services. The mathematical formulation representing virtual inertia is expressed as:

8 $${G}_{VI}\left(s\right)=\left(\frac{J}{1+{T}_{vi}s}\right)$$

where *J* denotes the system’s virtual inertia, ${T}_{vi}$ is the time constant that defines the system’s response, and s is the Laplace operator. This equation illustrates how the virtual inertia system reacts to sudden frequency variations, thereby helping to maintain stability and ensuring reliable power supply in isolated renewable-based microgrids. Built on this perspective, this study employs a VSG model based on the swing equation, which is made up of dual chief mechanisms: the first for inertia emulation and the second for damping properties emulation.

This study explores the implementation of three distinct control strategies for virtual inertia support: PID, fuzzy-PID, and VSC-FPID controllers. Each approach offers unique advantages in terms of dynamic performance and robustness. The comparative analysis evaluates their effectiveness in supporting virtual inertia and maintaining stable frequency regulation under diverse load and generation conditions.

### Conventional virtual inertia control based on PID controller

**Fig. 3:**
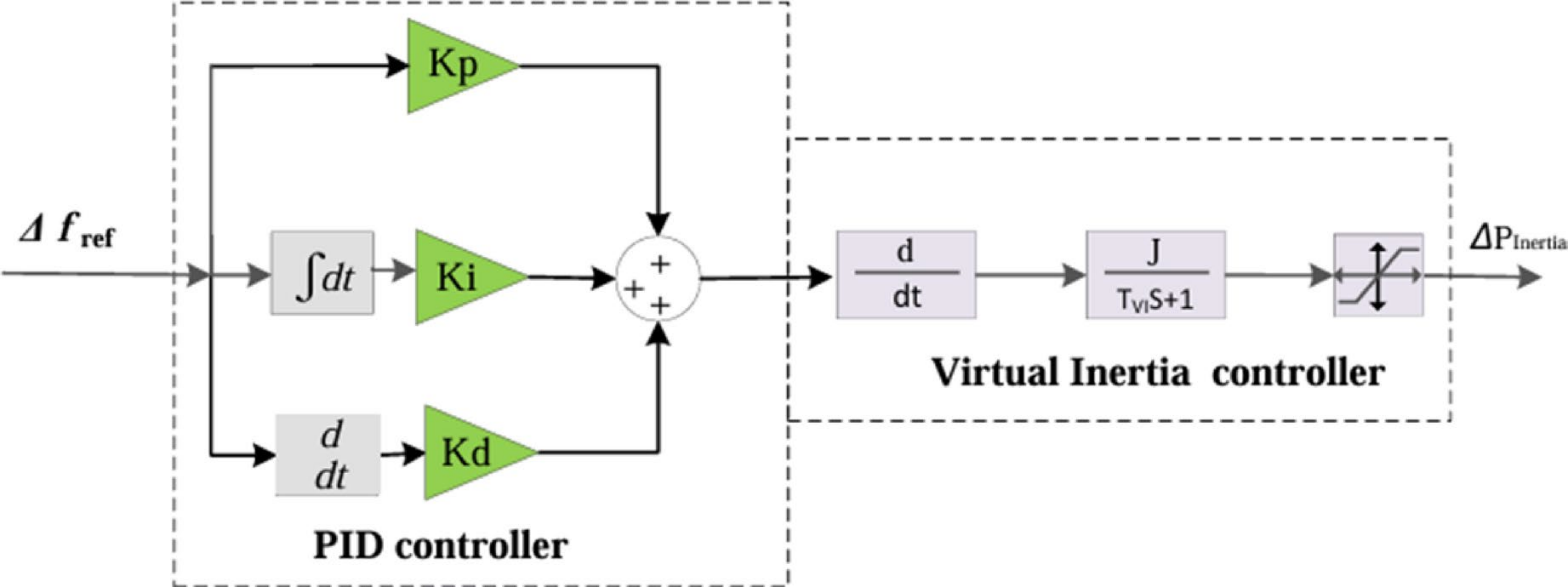
Structure of virtual inertia control based on a PID controller.

The PID controller remains one of the most extensively employed strategies for improving overall system performance, particularly in terms of stability, voltage regulation, dynamic response, and accuracy. The configuration of the proposed PID control scheme, employed to support virtual inertia control, is illustrated in Fig. 3. To achieve optimal performance, the tuning of the PID parameters is carried out using advanced optimization techniques. In this study, the PSO algorithm is utilized to identify the most effective gain values for the PID controller. The mathematical representation of the PID controller is provided in Eq. (9).

9 $${G\left(s\right)}_{PID}={K}_{P}+\frac{{K}_{i}}{s}+{K}_{d}s$$

### Virtual inertia control based on fuzzy-PID controller

**Fig. 4:**
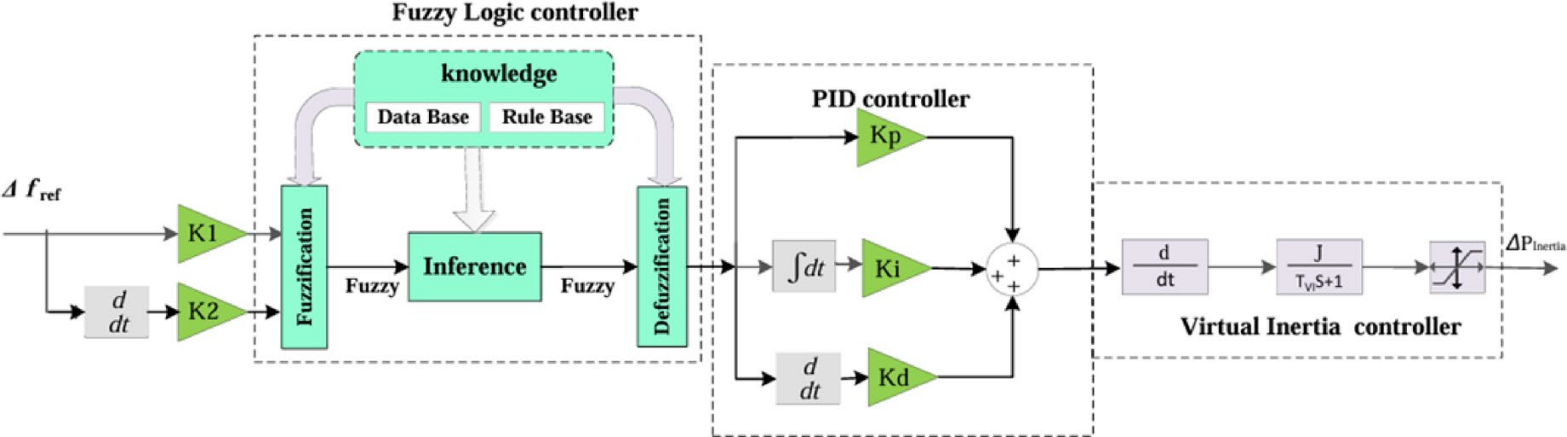
Schematic structure of the virtual inertia control system incorporating a fuzzy-PID controller.

**Fig. 5:**
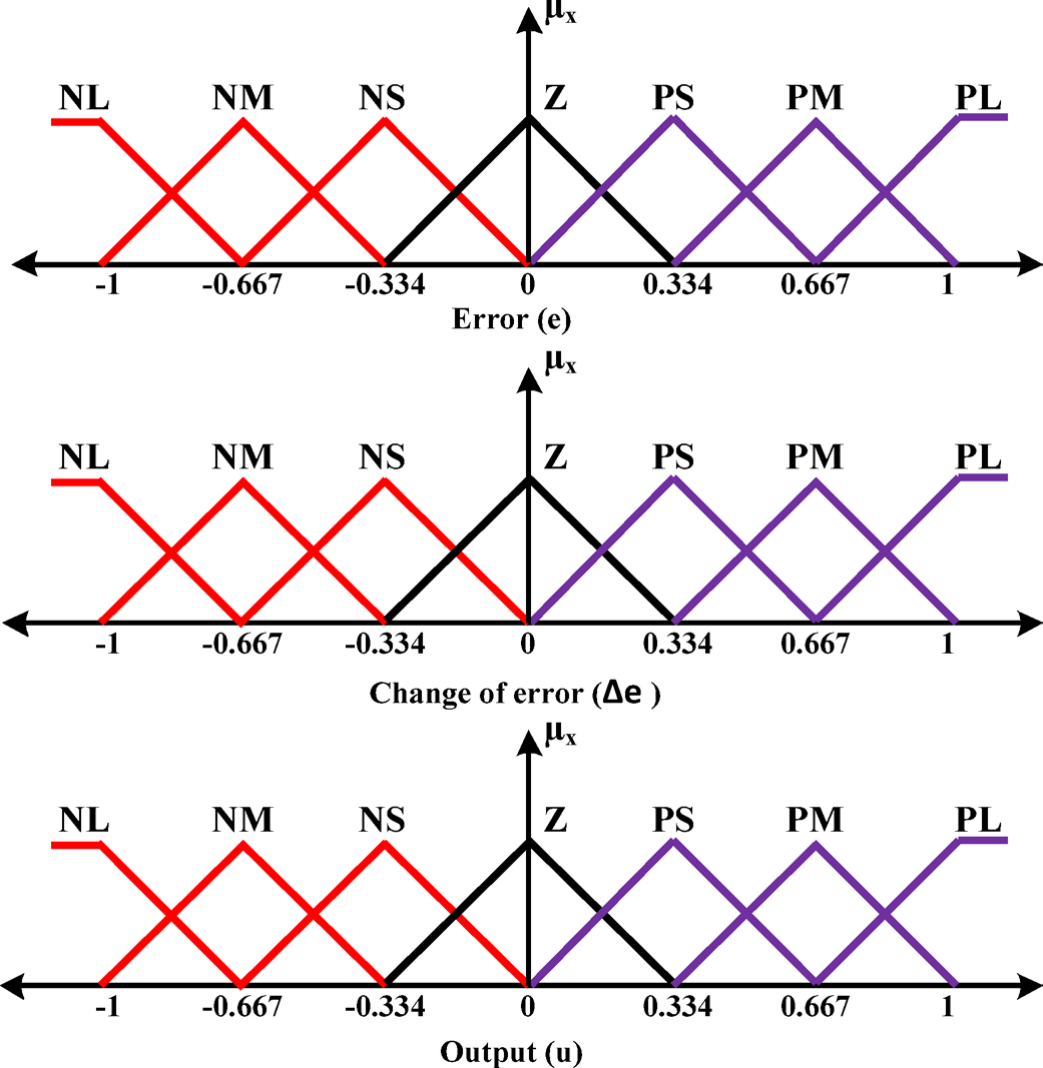
Membership functions.

The Fuzzy-PID controller is a rule-based control system that combines fuzzy logic with conventional PID actions. This controller architecture comprises four core components: fuzzification, a rule base, an inference engine, and a defuzzification unit. Fuzzification is responsible for transforming precise numerical inputs into fuzzy sets using linguistic terms. The rule base serves as a repository of expert knowledge, containing predefined rules that outline the appropriate response for each possible input condition. The inference mechanism evaluates the active rules based on the current input, generating fuzzy outputs accordingly. These outputs are then combined through an aggregation process to produce a final fuzzy output set, which is subsequently converted back into a crisp control signal through the defuzzification process. Defuzzification translates the fuzzy output into a precise numerical value that can be directly applied to the controlled system. The configuration of the proposed fuzzy-PID control scheme, employed to support virtual inertia control, is illustrated in Fig. 4. In the suggested fuzzy controller design, the variables: error (e), change in error (Δe), and controller output (u) are each represented by seven fuzzy sets. Figure 5 illustrates the membership functions assigned to these variables, categorized using seven linguistic labels: Positive Small (PS), Positive Medium (PM), and Positive Large (PL), Zero (ZE), Negative Large (NL), Negative Medium (NM), and Negative Small (NS). Table 2 presents the complete rule base that defines the operational logic of the fuzzy controller.

The output corresponding to each fuzzy rule is determined by applying either the minimum or product inference method to the input weights. For a given fuzzy output set, the aggregation process employs the maximum operator, denoted as ${\mu}_{i}$. Following the inference stage, the defuzzification process applies the weighted average technique to derive a crisp control output. As expressed in Eq. (10), the output is computed by considering *N* output membership functions, where θ denotes the centroid of each associated membership function.

10 $$u = \frac{{\mathop \sum \nolimits_{{i = 1}}^{N} \mu _{i} \theta _{i} }}{{\mathop \sum \nolimits_{{i = 1}}^{N} \mu _{i} }} = \xi \underset{\raise0.3em\hbox{$\smash{\scriptscriptstyle-}$}}{\theta }$$

11 $$\underset{\raise0.3em\hbox{$\smash{\scriptscriptstyle-}$}}{\theta } = \left[ {\begin{array}{*{20}c} {\theta _{1} } & {\theta _{2} } & {\theta _{3} } & \ldots & {\theta _{N} } \\ \end{array} } \right]^{T}$$

12 $$\xi ~~~ = \left[ {\begin{array}{*{20}c} {\xi _{1} } & {\xi _{2} } & {\xi _{3} } & \ldots & {\xi _{N} } \\ \end{array} } \right]$$

13 $$\xi _{i} = \frac{{\mu _{i} }}{{\mathop \sum \nolimits_{{i = 1}}^{N} \mu _{i} }}$$

**Table:** Base fuzzy rule for 7 membership functions.

e	Δe						
	NL	NM	NS	ZE	PS	PM	PL
NL	NL	NL	NL	NL	NM	NS	ZE
NM	NL	NL	NL	NM	NS	ZE	PS
NS	NL	NL	NM	NS	ZE	PS	PM
ZE	NL	NM	NS	ZE	PS	PM	PL
PS	NM	NS	ZE	PS	PM	PL	PL
PM	NS	ZE	PS	PM	PL	PL	PL
PL	ZE	PS	PM	PL	PL	PL	PL

### Proposed virtual inertia control based on VSC-FPID

**Fig. 6:**
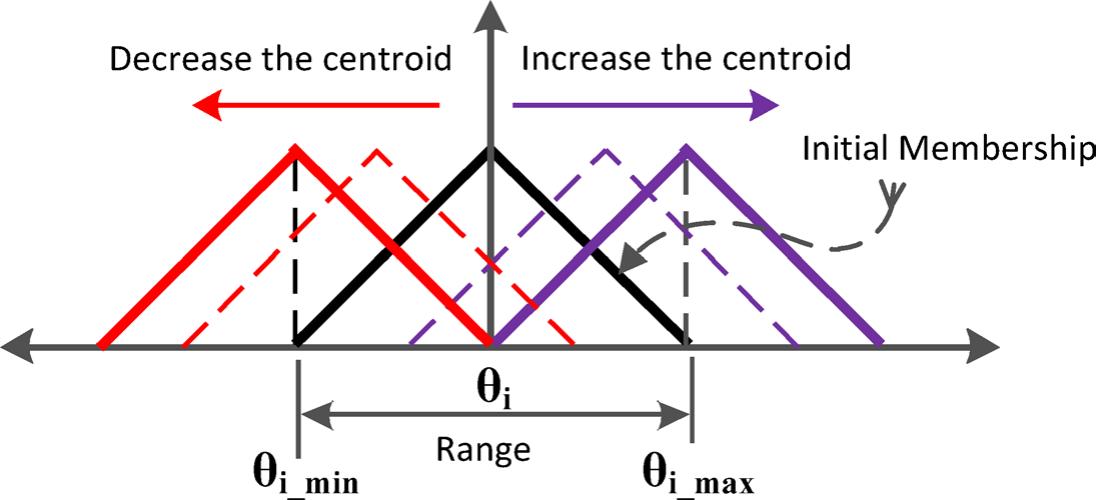
Adaptation of the membership centroid.

In conventional fuzzy logic controllers, the centroids of the output membership functions remain fixed. In contrast, the variable structure fuzzy controller dynamically adjusts these centroids in real-time, as illustrated in Fig. 6. This online adaptation process modifies the fuzzy output based on Eq. (10). The adaptation mechanism, responsible for tuning the output membership function centroids ${{\uptheta}}_{\mathrm{i}}$ during operation. The derivation of the adaptation law is outlined as follows:

14 $${\theta}_{i}=\stackrel{-}{{\theta}_{i}}sgn\left({\underset{\_}{e}}^{T}{\underset{\_}{P}}_{n}\right)+{\theta}_{i}\left(0\right).$$

15 $${e}_{k}={\Delta}f$$

16 $${\Delta}{e}_{k}={e}_{k}-{e}_{k-1}$$

17 $${\underset{\_}{e}}^{T}=\left[\begin{array}{cc}{e}_{k}&{\Delta}{e}_{k}\end{array}\right]$$

In^16,17^ and, the adaptation law applied to a power system stabilizer and the yaw control of a wind turbine is expressed by Eq. (14). The term $\stackrel{-}{{\theta}_{i}}$ represents a constant value reflecting the designer’s confidence in the initial selection of ${\theta}_{i}\left(0\right)$. Additionally, ${\underset{\_}{P}}_{n}$ denotes a Lyapunov positive definite matrix, while ${\underset{\_}{e}}^{T}$ refers to the error vector derived using Eqs. (14) through (16).

To mitigate chattering, the (sgn) function in Eq. (13) is substituted with a (sat) function, as presented in Eq. (18). By substituting Eq. (17) into Eq. (18), the final form of the adaptation law is derived, as shown in Eq. (18). Here, α and β are determined from the Lyapunov matrix. ${\upsilon}_{i}$ denotes a constant that quantifies the level of confidence in the initial value of ${\theta}_{i}\left(0\right)$

18 $${\theta}_{i}=\stackrel{-}{{\theta}_{i}}sat\left({\underset{\_}{e}}^{T}{\underset{\_}{P}}_{n}\right)+{\theta}_{i}\left(0\right).$$

19 $$\theta _{i} = \upsilon _{i} \mathop {e^{T} }\limits_{\_} P_{2} = \upsilon _{i} \left( {\alpha e_{k} + \beta \Delta e_{k} } \right) + \theta _{i} \left( 0 \right)\forall \in \{ 1,2, \ldots ,7\}$$

**Fig. 7:**
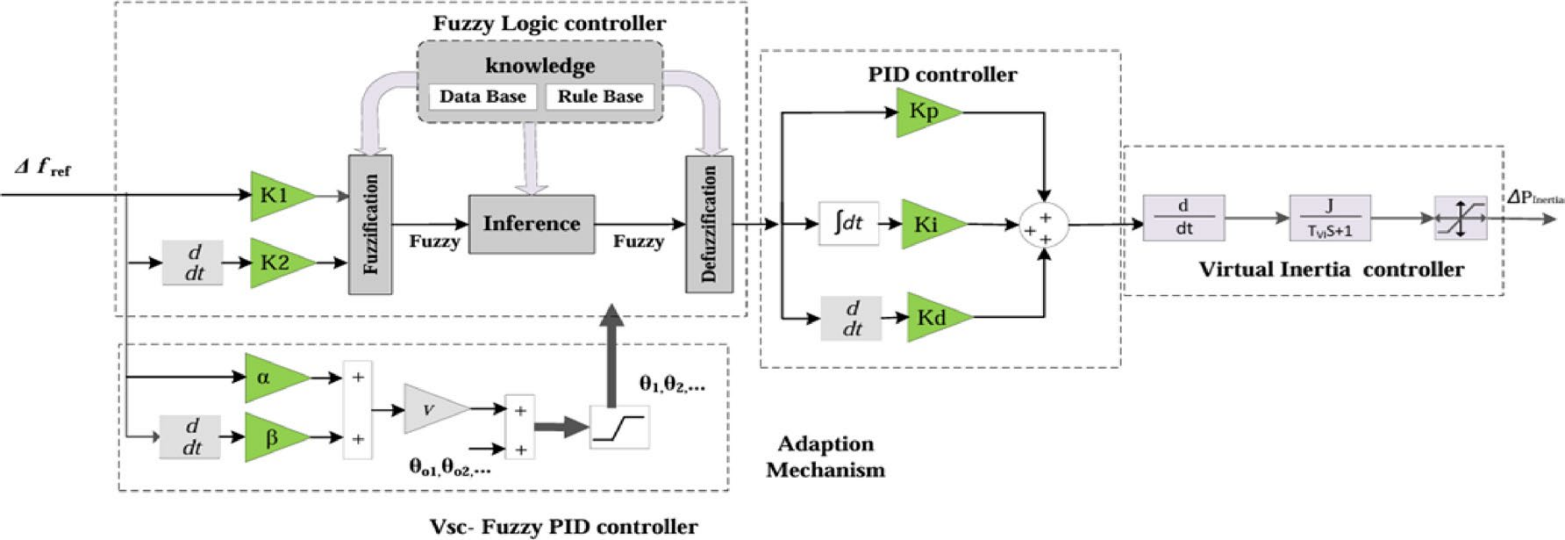
Structure of proposed virtual inertia control strategy based on VSC-fuzzy PID controller.

The design of adaptation laws using the Lyapunov approach typically relies on several simplifying assumptions, such as neglecting GRC and system saturation effects. As a more practical and effective alternative, meta-heuristic optimization techniques can be employed to tune the adaptation gains (α and β), while accounting for the inherent system nonlinearities. Figure 7 illustrates the architecture of the proposed virtual inertia control scheme, which integrates a VSC-based fuzzy PID controller.

As depicted in Fig. 7, the proposed control framework incorporates multiple tunable parameters, which are determined by the number of output membership functions, denoted by *N*. The parameters associated with the centroids of the output membership functions (*v*1, *v*2,…) are consolidated into a single variable to simplify the optimization process, ensuring a uniform adjustment across all centroids. Consequently, the final set of tuning parameters comprises seven key variables: the input gains of the fuzzy controller ($k1,k2$), the PID controller gains ($kp,ki,kd$), and the adaptation mechanism gains ($\alpha,\beta$). The PSO technique is employed to find the optimal values for these parameters. The PSO is a widely adopted and efficient optimization technique, particularly well-suited for handling nonlinear and complex systems. It is favored for its simplicity, fast computational performance, and rapid convergence, outperforming other algorithms such as genetic algorithms, ant colony optimization, and the aquila optimizer. In the PSO, each candidate solution represents a particle within the swarm, and the particles iteratively update their positions by balancing individual best experiences with the global best solution found by the entire swarm. This position-updating process is governed by two main equations, which are detailed in the following section.

In Eq. (20), the particle’s updated velocity is computed, while Eq. (21) defines the particle’s updated position. In these equations, ${{v}_{i}}^{k}\mathrm{a}\mathrm{n}\mathrm{d}{{v}_{i}}^{k+1}$ represent the particle’s current and updated velocities, respectively. The terms ${{g}_{best}\mathrm{a}\mathrm{n}\mathrm{d}P}_{best,i}$ denote the global best position identified by the swarm and the personal best position of the particle itself. The parameters $W,{c}_{1},{c}_{2}$ are the inertia weight, the cognitive (individual) factor, and the social (swarm) factor, respectively. Additionally, ${r}_{1}\mathrm{a}\mathrm{n}\mathrm{d}{r}_{2}$ are random numbers uniformly distributed between [0, 1], and ${{x}_{i}}^{k}\mathrm{a}\mathrm{n}\mathrm{d}{{x}_{i}}^{k+1}$ represent the particle’s current and updated positions. Each particle in the swarm is evaluated using a specified objective function outlined in Eq. (21). This objective function comprises three distinct components:

1. The Integral of Time-weighted Absolute Error (ITAE) term, which minimizes the time-weighted absolute frequency error, promotes faster system response by imposing higher penalties on errors that persist over time.
2. The second term aims to suppress frequency oscillations by minimizing overshoot.
3. The third term focuses on reducing RoCoF to enhance system stability during disturbances.

The weighting factors ${K}_{1},{K}_{2},and{K}_{3}$ in the objective function serve to balance the contributions of each performance criterion while also ensuring proper unit consistency^4^. The optimization is carried out under the constraint conditions defined by the inequality expressions in Eq. (22), with the resulting optimal controller parameters listed in Table 3.

20 $${{v}_{i}}^{k+1}=W{{v}_{i}}^{k}+{c}_{1}{r}_{1}\left({P}_{best,i}-{{x}_{i}}^{k}\right)+{c}_{2}{r}_{2}\left({g}_{best}-{{x}_{i}}^{k}\right)$$

21 $${{x}_{i}}^{k+1}={{x}_{i}}^{k}+{{v}_{i}}^{k+1}$$

22 $$OF = \min \left[ {K_{1} \smallint _{0}^{t} t\left\lceil f \right\rceil dt + K_{2} \max \left( {\Delta f,0} \right) + K_{3} \frac{{\Delta f}}{{\Delta t}}} \right]$$

Subject to:

**Table 3:** Optimal parameters of different controllers that are optimized by PSO.

Controller type	Optimization gains	Different controller’s gains are optimized by PSO for Virtual inertia						
		${\mathrm{K}}_{1}$	${\mathrm{K}}_{2}$	$\alpha$	$\beta$	${\mathrm{K}}_{\mathrm{p}}$	${\mathrm{K}}_{i}$	${\mathrm{K}}_{\mathrm{d}}$
Optimal PID	${\mathrm{K}}_{\mathrm{p}},{\mathrm{K}}_{i}and{\mathrm{K}}_{\mathrm{d}}$	–	–	–	–	− 33.4151	− 17.1984	− 14.663
Optimal FPID	${{\mathrm{K}}_{1},{\mathrm{K}}_{2},\mathrm{K}}_{\mathrm{p}},{\mathrm{K}}_{i},{and\mathrm{K}}_{\mathrm{d}}$	− 1.9996	-2.3281	–	–	4.9999	0.4551	5.1090
Optimal VSC-FPID	${{\mathrm{K}}_{1},{\mathrm{K}}_{2},\alpha,\beta,\mathrm{K}}_{\mathrm{p}},{\mathrm{K}}_{i},{\mathrm{a}\mathrm{n}\mathrm{d}\mathrm{K}}_{\mathrm{d}}$	− 1.6671	− 3.7028	− 1.9897	− 0.3013	17.1760	2.4372	0.6564

23 $$\begin{gathered} k_{p}^{{min}} \leqslant {k_p} \leqslant k_{p}^{{max}} \hfill \\ k_{d}^{{min}} \leqslant {k_d} \leqslant k_{d}^{{max}} \hfill \\ k_{i}^{{min}} \leqslant {k_i} \leqslant k_{i}^{{max}} \hfill \\ k_{1}^{{min}} \leqslant {k_1} \leqslant k_{1}^{{max}} \hfill \\ k_{2}^{{min}} \leqslant {k_2} \leqslant k_{2}^{{max}}{\alpha ^{min}} \leqslant \alpha \leqslant {\alpha ^{max}} \hfill \\ {\beta ^{min}} \leqslant \beta \leqslant {\beta ^{max}} \hfill \\ \end{gathered}$$

## Simulation results and discussion

To rigorously evaluate the performance of the proposed VSC-FPID controller for virtual inertia support, extensive simulation studies were conducted in the MATLAB/SIMULINK environment. The controller’s parameters were optimally tuned using the PSO algorithm to ensure superior dynamic response. The proposed VSC-FPID controller is evaluated under seven distinct scenarios covering a comprehensive set of operational conditions, including step load changes, stochastic load variations, industrial and residential demand profiles, wind and solar PV fluctuations, and reduced system inertia. For each case, comparative performance with conventional PID and fixed-structure fuzzy PID (FPID) controllers is analyzed to demonstrate the robustness and effectiveness of the proposed approach under diverse disturbances and system nonlinearities.

**Fig. 8:**
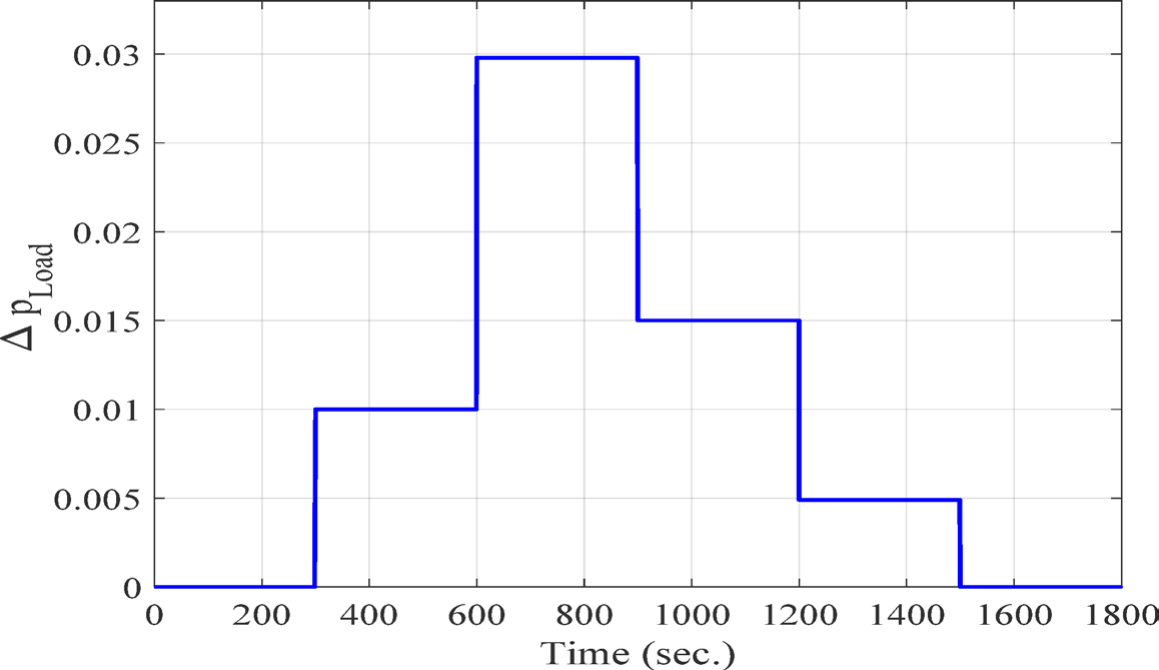
Load power variations for Case (1).

**Fig. 9:**
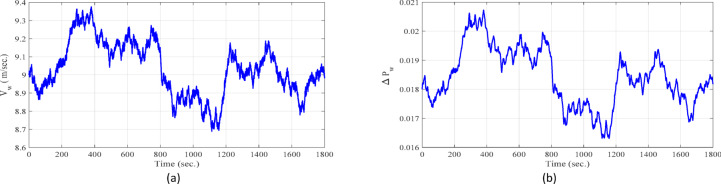
Response of the wind generation system (**a**): Wind speed, and (**b**) Wind power variation.

**Fig. 10:**
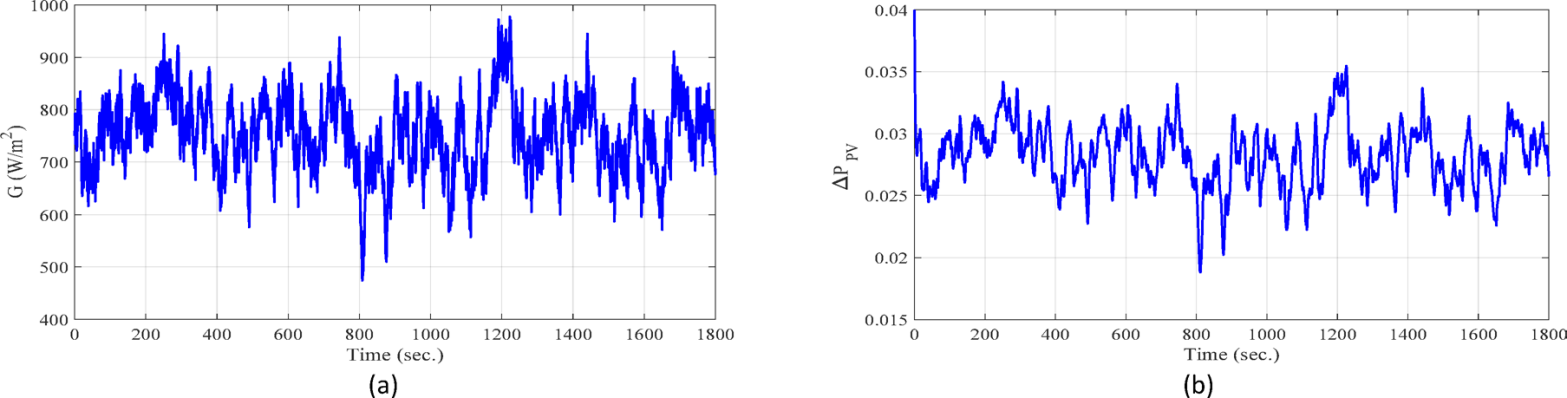
Response of the solar PV system (**a**): Solar irradiation profile and (**b**) Solar PV power profile.

**Fig. 11:**
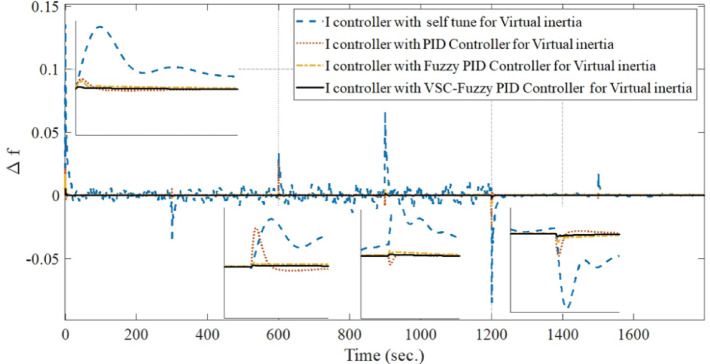
System frequency deviation under Case 1 conditions.

**Case 1: **In this case, a cascade of step load change is applied, as illustrated in Fig. 8, along with variations in wind power, illustrated in Fig. 9. The wind power generation connects at 600 s and remains until the end of the simulation. Meanwhile, the solar PV generation, shown in Fig. 10, is connected at the beginning of the simulation (0 s) and is disconnected at 1200 s. The system’s frequency deviation with virtual inertia control, implemented using different controllers; FPID, PID, and the proposed VSC-FPID controller, is presented in Fig. 11. Simulation results reveal that the FPID controller reduces frequency deviations more effectively than the conventional PID controller; however, it demonstrates a slower convergence rate, requiring more time to achieve steady-state conditions. In contrast, the suggested VSC-FPID controller achieves superior performance by reducing both the frequency deviations and the settling time. Under smaller load disturbances, the settling time differences between controllers become evident, with the VSC-FPID controller achieving the fastest response. Similarly, for larger disturbances, the proposed controller outperforms the others by settling significantly faster, highlighting its enhanced effectiveness. Furthermore, in terms of frequency nadir, the VSC-FPID controller demonstrates clear improvement, ensuring better frequency stability under both minor and severe disturbances when compared to the PID and FPID controllers. According to Table 4, the I controller with VSC-Fuzzy PID for virtual inertia outperforms all other controllers by providing the minimum overshoot and undershoot with satisfactory settling times.

**Table 4:** Dynamic response of all control techniques for case 1.

Time period (in seconds)		I controller with self-tune for Virtual inertia	I controller with PID for Virtual inertia	I controller with Fuzzy PID for Virtual inertia	I controller with VSC-Fuzzy PID for Virtual inertia
From zero to 12 s	Undershoot	0	0.0031024	0	4.4508e-05
	Overshoot	0.13533	0.021987	0.016688	0.005319
	Settling Times	10.623	5.0778	9.0493	6.6327
From 300 to 310 s.	Undershoot	0.036871	0.0007761	0.0025388	0.00096634
	Overshoot	0.0043974	0.0059718	0.00041833	3.3655e-06
	Settling Times	9.6128	8.3691	9.5743	9.4538
From 600 to 612 s.	Undershoot	0.0011648	0.0030421	3.1108e-07	5.8704e-05
	Overshoot	0.033781	0.027468	0.0025554	0.0010288
	Settling Times	11.788	5.401	11.312	11.006
From 900 to 910 s.	Undershoot	0.0095149	0.0082913	0.00030396	0.00015683
	Overshoot	0.066886	0.0010311	0.0048853	0.0018463
	Settling Times	9.7494	8.4966	9.7799	9.7272
From 1200 to 1210 s.	Undershoot	0.085241	0.025243	0.009601	0.0031479
	Overshoot	0.0059663	0.0029495	0.00029678	9.1805e-05
	Settling Times	9.3661	5.5812	9.1236	8.8869
From 1500 to 1512 s.	Undershoot	0.00047427	0.0027716	7.0042e-05	2.1687e-05
	Overshoot	0.017165	0.00033485	0.001198	0.00048854
	Settling Times	9.3562	5.0825	9.1293	10.712

**Fig. 12:**
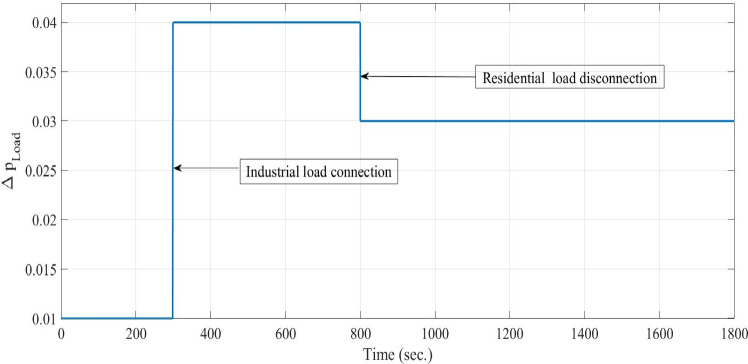
Variation in residential and industrial loads.

**Fig. 13:**
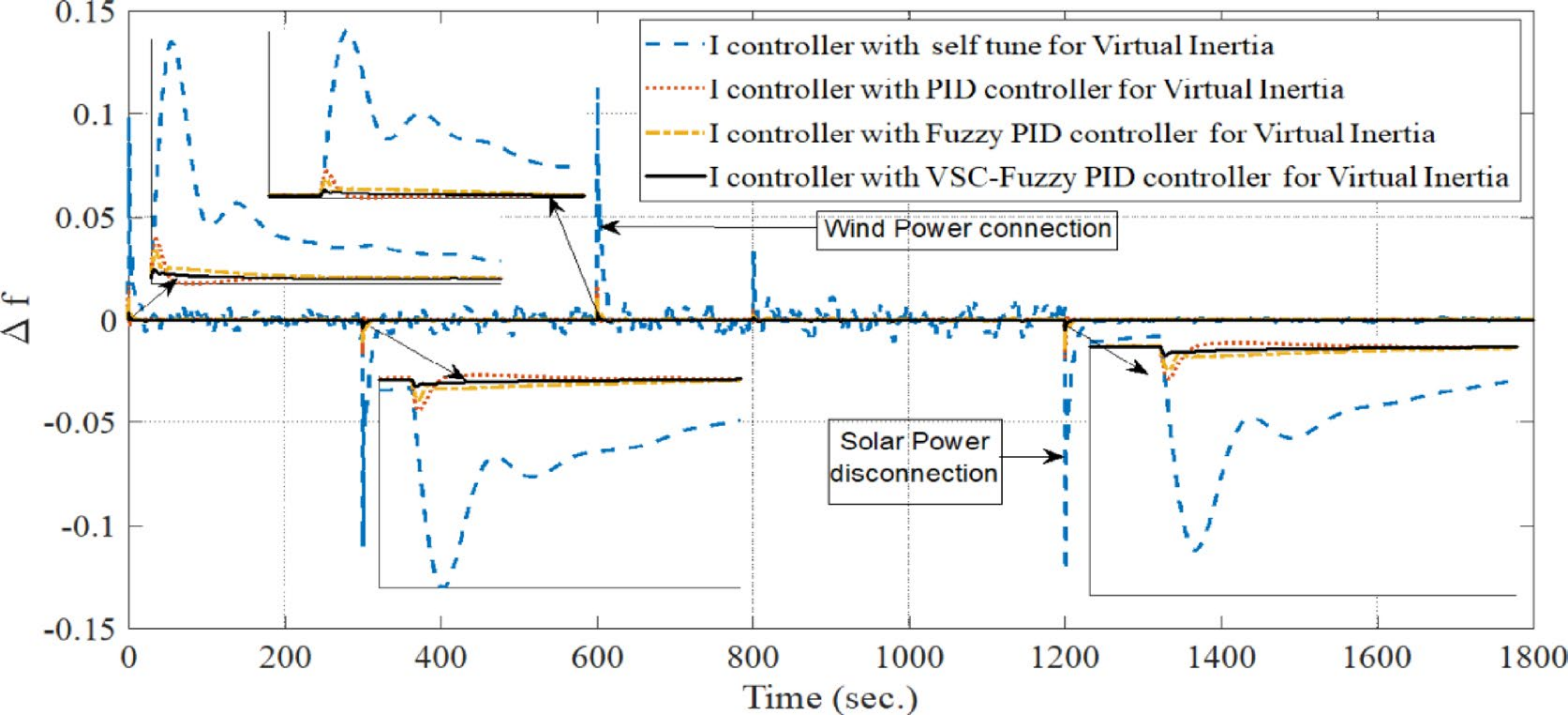
System frequency deviation under Case 2 conditions.

**Case 2: **Modern power systems rely heavily on RESs, particularly wind and solar (PV), which are highly weather-dependent and introduce significant power fluctuations. These non-dispatchable sources require effective control strategies to regulate dispatchable thermal units and mitigate their impact on system frequency. In short-term simulations, solar irradiance fluctuates randomly, while temperature is assumed to be constant. Wind power depends on wind speed, turbine characteristics, and control mechanisms, with wind speed modeled as white noise. The PV and wind power generation profiles, depicted in Figs. 9 and 10, are implemented under the same operational conditions as those in Case 1. Additionally, a random load variation—representing industrial and residential demand—increases by 4% at 300 s and then decreases to 3% at 800 s, as illustrated in Fig. 12. Figure 13 illustrates the system’s frequency deviation under virtual inertia control implemented using PID, FPID, and the proposed VSC-FPID controllers. As observed, the PID and FPID controllers yield higher frequency deviations and increased oscillatory behavior. In contrast, the proposed VSC-FPID controller significantly improves dynamic performance, effectively minimizing both deviation amplitude and oscillations. Moreover, due to sudden PV fluctuations, the PID and FPID controllers exhibit higher frequency oscillations, while the proposed controller effectively maintains frequency stability under simultaneous PV and wind variations.

**Fig. 14:**
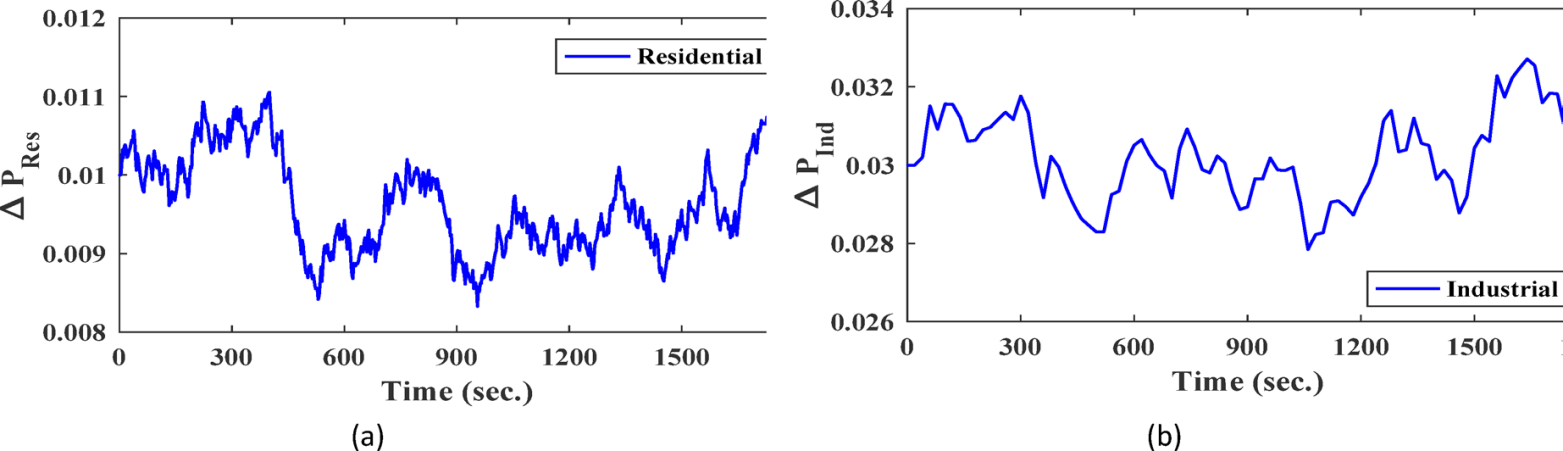
Load Variations (**a**): Residential load variations, (**b**) industrial load variations and (**c**) Incorporating random load fluctuations.

**Fig. 15:**
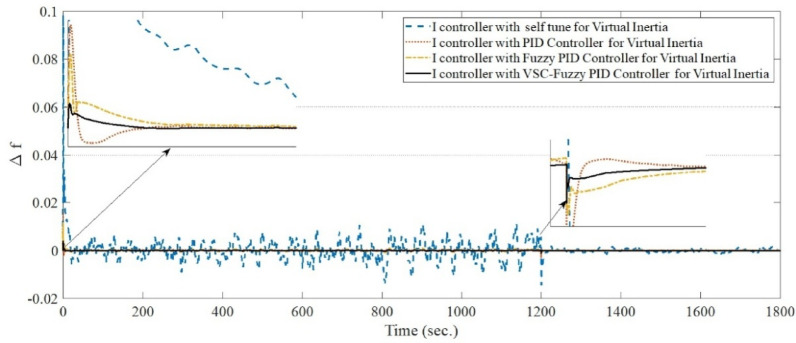
System frequency deviation under Case 3 conditions.

**Case 3: **Indeed, residential loads are generally smaller in scale but tend to exhibit frequent and irregular fluctuations, while industrial loads are larger and have a more pronounced impact on system frequency when switched on or off. Figure 14 illustrates the variations in both residential and industrial load profiles considered in this study. Specifically, the industrial load is abruptly introduced at 600 s and disconnected at 1200 s. As a result, the system initially operates under residential loading conditions, followed by sudden integration and subsequent removal of the industrial load. The industrial loads introduce more significant frequency deviations, requiring precise control to maintain system stability and reliability. On the other side, the PV and wind power generation profiles, depicted in Figs. 9 and 10, are implemented under the same operational conditions as those in Case 1. The frequency variation of the studied system with virtual inertia control, using FPID, PID, and the proposed VSC-FPID controllers, is shown in Fig. 15. The findings demonstrate that when the industrial load is abruptly added, the system frequency drops, and when it is removed, the frequency rises accordingly. The suggested controller outperforms both conventional PID and FPID controllers by achieving restoring frequency more quickly, with reduced deviations and faster settling times. Additionally, the proposed controller effectively manages larger load disturbances, highlighting its enhanced capability in maintaining system stability.

**Fig. 16:**
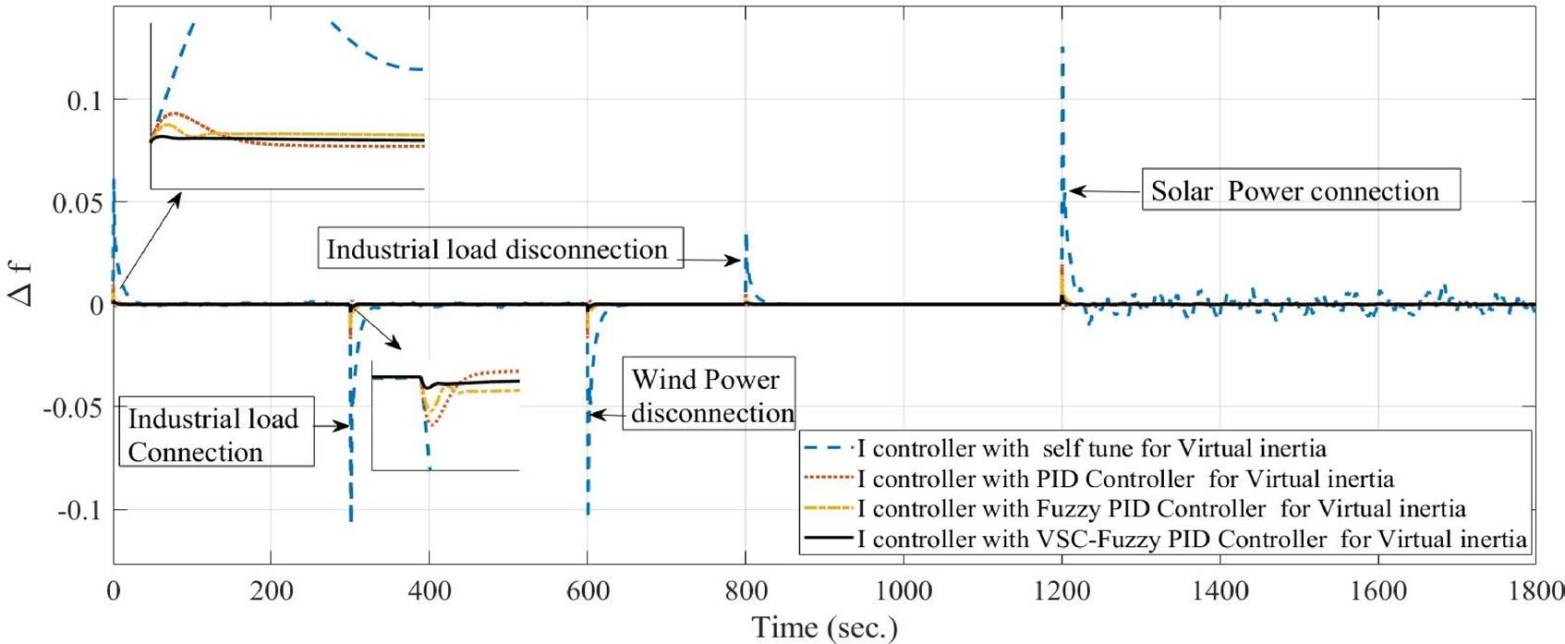
System frequency deviation under Case 4 conditions.

**Case 4: **In this case study, a detailed simulation is conducted to evaluate and compare the performance of virtual inertia control based on the proposed VSC-FPID controller against conventional PID and FPID controllers. The comparison is conducted under a combination of realistic operating conditions, including random load variations (as shown in Fig. 12), wind power fluctuations (as shown in Fig. 9) applied from the start of the simulation to 600 s, and solar PV power fluctuations (as shown in Fig. 10) introduced from 1200 s until the end of the simulation. The system’s frequency deviation, shown in Fig. 16, highlights that the suggested VSC-FPID controller achieves Outstanding performance in contrast to the PID and FPID controllers. Specifically, the proposed controller significantly reduces both overshoot and undershoot while providing faster settling time, demonstrating its enhanced capability to maintain frequency stability under dynamic operating conditions.

**Fig. 17:**
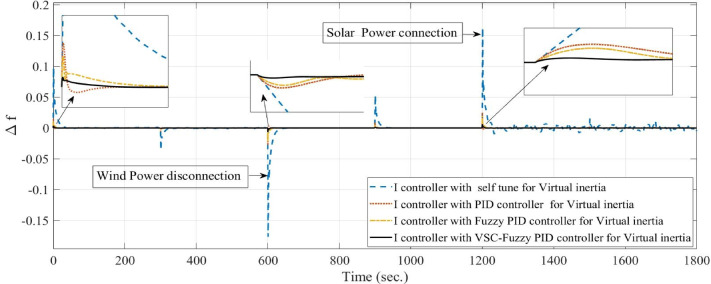
System frequency deviation under Case 5 conditions.

**Case 5: **In this case study, the performance of virtual inertia control using the proposed VSC-FPID controller is in comparison with conventional PID and FPID controllers under a different set of operating conditions. A cascade of step load changes, as shown in Fig. 8, is applied to emulate realistic demand fluctuations. Concurrently, wind power generation fluctuates from the beginning of the simulation up to 600 s (Fig. 9), while solar PV generation introduces variability from 1200 s to the end of the simulation period (Fig. 10). The resulting system frequency deviations, presented in Fig. 17, confirm that the suggested virtual inertia control depended on VSC-FPID controller consistently outperforms both the traditional PID and the FPID controllers in terms of frequency stability and dynamic response. The suggested controller achieves reduced overshoot and undershoot, along with faster frequency stabilization, confirming its superior effectiveness in maintaining system stability under complex and variable operating conditions. According to Table 5, the I controller integrated with VSC-Fuzzy PID–based virtual inertia demonstrates the best overall performance by achieving minimal overshoot and undershoot with satisfactory settling times.

**Table 5:** Dynamic response of all control techniques for case 5.

Time period (in seconds)		I controller with self-tune for Virtual inertia	I controller with PID for Virtual inertia	I controller with Fuzzy PID for Virtual inertia	I controller with VSC-Fuzzy PID for Virtual inertia
From zero to 15 s	Undershoot	0	0.0018234	0	7.1688e-05
	Overshoot	0.097487	0.015221	0.010566	0.0034997
	Settling Times	13.749	5.741	12.293	9.6467
From 300 to 310 s.	Undershoot	0.035454	0.0055732	0.002975	0.0011097
	Overshoot	0.00027753	0.00066171	0.00016618	4.7219e-06
	Settling Times	9.409	5.2548	9.3483	9.0905
From 600 to 670 s.	Undershoot	0.17664	0.027692	0.022067	0.0067886
	Overshoot	0.00018998	0.0033204	1.5054e-06	0.00015019
	Settling Times	28.604	5.6246	14.834	15.453
From 900 to 920 s.	Undershoot	1.5756e-13	0.00099967	5.6023e-05	4.5115e-05
	Overshoot	0.052929	0.0083227	0.0048953	0.001764
	Settling Times	17.526	5.6255	14.39	9.1286
From 1200 to 1220 s.	Undershoot	4.7789e-14	-0.0032387	8.2407e-05	0.00020169
	Overshoot	0.16194	0.025323	0.019746	0.0061485
	Settling Times	16.496	5.5837	13.736	17.185

**Fig. 18:**
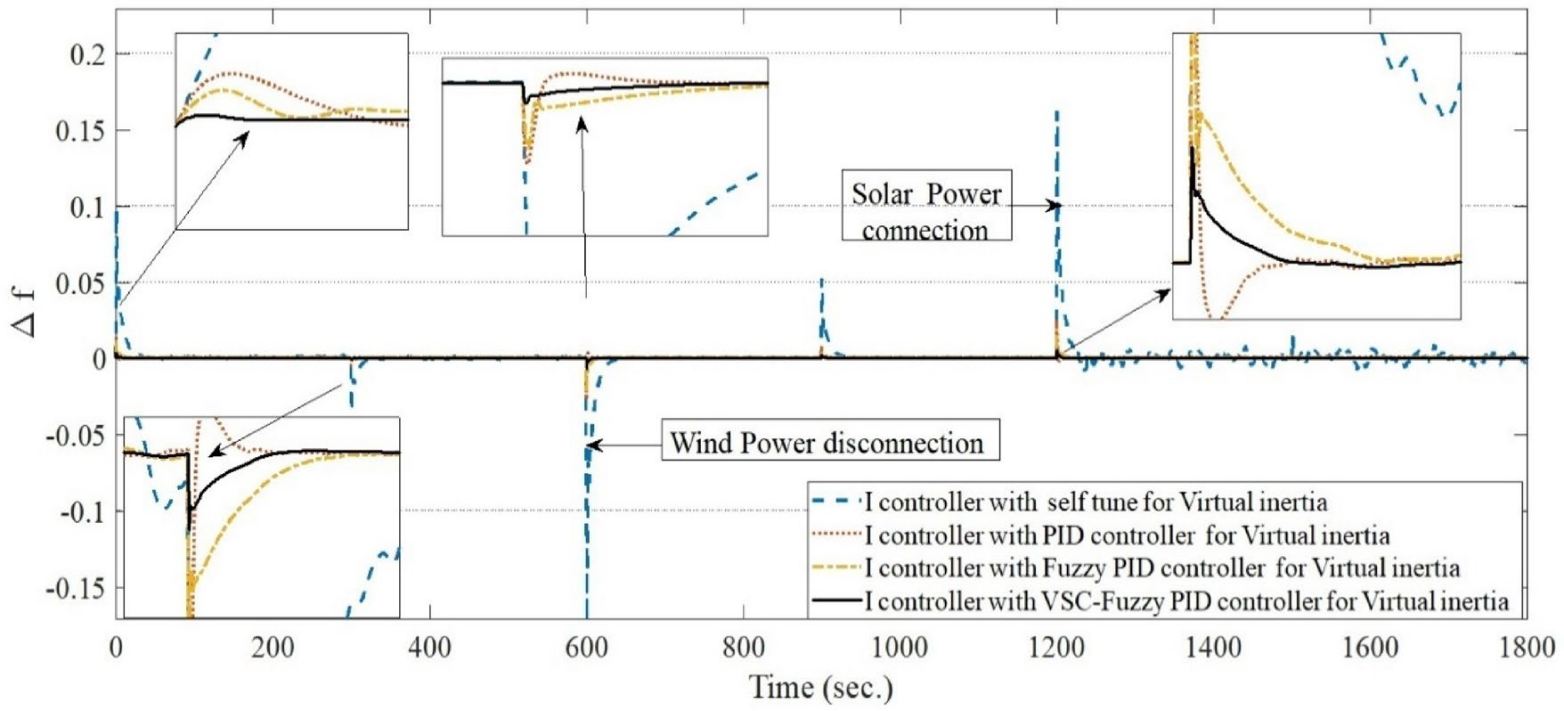
System frequency deviation under Case 6 conditions.

**Case 6: **In this case study, the performance of the proposed virtual inertia control using the VSC-FPID controller is evaluated and compared with the conventional PID and FPID controllers under more challenging operating conditions. A cascade of step load changes is applied, as depicted in Fig. 8, alongside fluctuating wind power generation (shown in Fig. 9) from 0 to 600 s, and varying solar PV power generation (shown in Fig. 10) from 1200 s until the end of the simulation. Additionally, system uncertainties are introduced by reducing the system’s inertia to 85% of its nominal value, simulating a low-inertia scenario. The system frequency deviation, presented in Fig. 18, confirms the superior performance of the proposed VSC-FPID controller. In comparison with the PID and FPID controllers, the suggested controller achieves lower overshoot and undershoot, along with significantly faster frequency recovery, demonstrating its enhanced robustness and adaptability in handling both severe disturbances and system uncertainties.

**Case 7: **Sever-Case Operating Scenario (Simultaneous Load/RES Fluctuations and System Uncertainties): To further assess the robustness of the proposed VSC-FPID controller, this case study investigates a severe-case operating scenario involving simultaneous load variations, RES disturbances, and severe system uncertainties. Figure 14 illustrates the temporal variations of the residential and industrial load profiles considered in this case. The objective of this case is to examine the dynamic frequency response of the microgrid under extreme operating conditions that are representative of highly stressed, low-inertia renewable-dominated systems.

**Fig. 19:**
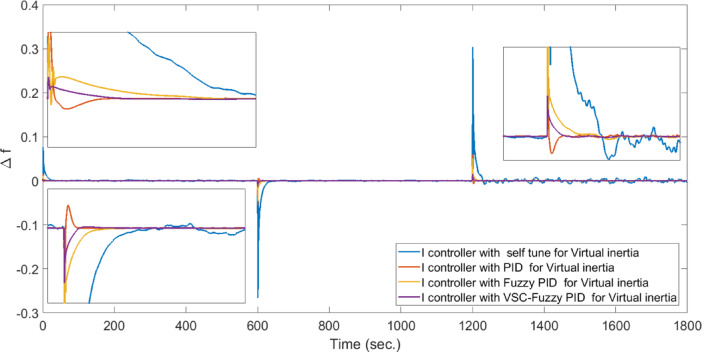
System frequency deviation under Case 7 conditions.

In this case, sudden disturbances are introduced in the converter-interfaced renewable generation units. As shown in Fig. 19, the wind power source is abruptly disconnected at approximately 600 s, followed by the connection of the solar PV source at around 1200 s. To emulate a critically low-inertia operating condition, the equivalent system inertia is reduced by 40% from its nominal value. In addition, the virtual inertia control gain is also reduced by 40%, reflecting practical constraints and uncertainties that may arise during real-world microgrid operation. The system performance is comparatively evaluated using four virtual inertia control strategies: the self-tuning controller, conventional PID, fuzzy PID (FPID), and the proposed variable-structure fuzzy PID (VSC-FPID) controller.

The simulation results reveal substantial differences in the dynamic performance of the investigated control strategies. Following the sudden wind power disconnection, the self-tuning controller exhibits a severe frequency deviation accompanied by large oscillations and a prolonged settling time, indicating insufficient damping capability under reduced inertia conditions. In contrast, the conventional PID controller provides a noticeable improvement by reducing frequency oscillations and shortening the settling time, although residual oscillations remain evident. The fuzzy PID controller further enhances the dynamic response by improving damping characteristics and significantly reducing both the maximum overshoot and settling time, demonstrating its ability to handle system nonlinearities and parameter uncertainties.

Among all control strategies, the proposed VSC-FPID controller achieves the best overall performance. It exhibits the smallest frequency deviation, minimal oscillatory behavior, and the fastest restoration of the nominal frequency following the wind power disconnection, highlighting its superior robustness and adaptive capability under severe inertia reduction. A similar trend is observed during the solar PV connection event at 1200 s. The self-tuning controller shows a sharp frequency rise followed by sustained oscillations, reflecting poor resilience to abrupt RES variations. Meanwhile, both the PID and FPID controllers deliver smoother and more regulated responses with improved transient stability. Nevertheless, the VSC-FPID controller consistently outperforms the other approaches by achieving the minimum frequency deviation and the shortest settling time.

Overall, the combined reduction of system inertia and virtual inertia gain by 40% significantly intensifies transient frequency deviations and aggravates system dynamics. While the conventional PID controller maintains an acceptable level of stability, and the fuzzy PID controller offers enhanced damping and operational flexibility, the proposed VSC-FPID controller proves to be the most effective solution. Its ability to rapidly restore frequency with negligible oscillations makes it particularly well-suited for low- or variable-inertia microgrids with high penetration of renewable energy sources and severe operating uncertainties.

To further substantiate the effectiveness of the proposed VSC-FPID controller, a comprehensive comparative analysis has been conducted against conventional PID and fixed-structure fuzzy PID controllers under a wide range of operating conditions. These conditions include step and stochastic load variations, intermittent wind and solar power fluctuations, residential and industrial demand profiles, reduced system inertia, and severe worst-case disturbances. Across all investigated scenarios, the VSC-FPID controller consistently demonstrates superior dynamic performance in terms of reduced frequency overshoot and undershoot, faster settling time, and improved damping of oscillations. Notably, under extreme low-inertia and high-renewable penetration conditions, the adaptive variable-structure mechanism enables the proposed controller to maintain robust frequency regulation where conventional approaches exhibit degraded performance. These results confirm the effectiveness of the proposed VSC-FPID controller in addressing the identified gaps and highlight its suitability for practical application in renewable-dominated, low-inertia microgrids.

## Conclusion

This paper presented an efficient virtual inertia control strategy for low-inertia microgrids using the proposed VSC-FPID controller. The proposed controller is optimally tuned using the particle swarm optimization algorithm, enhancing its responsiveness and robustness. The success of the proposed VSC-FPID controller-based virtual inertia control highlighted the potential of hybrid intelligent control structures in handling complex system dynamics and nonlinearities. Through extensive simulations under various loading conditions and system nonlinearities, the proposed VSC-FPID controller delivered notable enhancements in frequency regulation performance over traditional PID and fuzzy-PID controllers. It demonstrated enhanced transient performance and resilience, with faster stabilization and reduced frequency deviations under uncertain RES generation and load changes. Notably, the proposed approach delivered up to 60% better performance than the nearest alternative. These findings validate the proposed VSC-FPID controller as an effective solution for providing virtual inertia support in modern low-inertia microgrids. Future work may extend this approach to multi-agent distributed systems, incorporate demand-side management, or explore hardware-in-the-loop validation for deployment in real-world microgrid platforms.

## Data Availability

All data generated or analyzed during this study are included in this article.
